# Artificial intelligence in mobile health for skin cancer diagnostics at home (AIM HIGH): a pilot feasibility study

**DOI:** 10.1016/j.eclinm.2023.102019

**Published:** 2023-05-25

**Authors:** Anna M. Smak Gregoor, Tobias E. Sangers, Just AH. Eekhof, Sydney Howe, Jeroen Revelman, Romy JM. Litjens, Mohammed Sarac, Patrick JE. Bindels, Tobias Bonten, Rik Wehrens, Marlies Wakkee

**Affiliations:** a Department of Dermatology, Erasmus MC Cancer Institute, University Medical Center Rotterdam, the Netherlands; b Department of Public Health and Primary Care, Leiden University Medical Centre, Leiden, the Netherlands; c School of Health Policy and Management, Erasmus University, Rotterdam, the Netherlands; d General Practice, Erasmus MC Rotterdam, Rotterdam, the Netherlands

**Keywords:** Artificial intelligence, Skin cancer, Mobile health, Primary care, General practitioners, Convolutional neural network

## Abstract

**Background:**

Artificial intelligence (AI)-based mobile phone apps (mHealth) have the potential to streamline care for suspicious skin lesions in primary care. This study aims to investigate the conditions and feasibility of a study that incorporates an AI-based app in primary care and evaluates its potential impact.

**Methods:**

We conducted a pilot feasibility study from November 22nd, 2021 to June 9th, 2022 with a mixed-methods design on implementation of an AI-based mHealth app for skin cancer detection in three primary care practices in the Netherlands (Rotterdam, Leiden and Katwijk). The primary outcome was the inclusion and successful participation rate of patients and general practitioners (GPs). Secondary outcomes were the reasons, facilitators and barriers for successful participation and the potential impact in both pathways for future sample size calculations. Patients were offered use of an AI-based mHealth app before consulting their GP. GPs assessed the patients blinded and then unblinded to the app. Qualitative data included observations and audio-diaries from patients and GPs and focus-groups and interviews with GPs and GP assistants.

**Findings:**

Fifty patients were included with a median age of 52 years (IQR 33.5–60.3), 64% were female, and 90% had a light skin type. The average patient inclusion rate was 4–6 per GP practice per month and 84% (n = 42) successfully participated. Similarly, in 90% (n = 45 patients) the GPs also successfully completed the study. GPs never changed their working diagnosis, but did change their treatment plan (n = 5) based on the app's assessments. Notably, 54% of patients with a benign skin lesion and low risk rating, indicated that they would be reassured and cancel their GP visit with these results (p < 0.001).

**Interpretation:**

Our findings suggest that studying implementation of an AI-based mHealth app for detection of skin cancer in the hands of patients or as a diagnostic tool used by GPs in primary care appears feasible. Preliminary results indicate potential to further investigate both intended use settings.

**Funding:**

SkinVision B.V.

**Research in context**

**Evidence before this study**

While previous literature describes the potential of artificial intelligence (AI)-based apps for skin cancer in a sterile research setting, there is still a knowledge gap on the actual impact once implemented in primary care. We searched PubMed for articles published between 01-01-2011 and 30-01-2023 using the search terms artificial intelligence and skin cancer, and identified 928 articles which were screened for relevance. A recent systematic review, that focused on evidence for implementation of AI-based algorithms for early detection of skin cancer in a primary care setting, found that there was insufficient evidence of efficacy for widespread implementation to be recommended.

**Added value of this study**

In this pilot feasibility study, we focused on the feasibility of a study investigating the implementation of an AI-based mHealth app in the hands of the patient and as a diagnostic tool for general practitioners (GPs). Our main findings were that studying the implementation of an AI-based mHealth app at either point in the primary care pathway is feasible and we identified several important barriers and facilitators for a larger study. Additionally, we found that a significant number of patients with a benign skin lesion and a low risk rating from the app indicated that with this result they would be reassured to stay at home and cancel their GP visit. Showing potential for AI-based applications to help diminish some of the burden that GPs in many countries face. We found no negative effect on the diagnostic accuracy of GPs, however the app did sometimes lead to a change in their treatment plan.

**Implications of all the available evidence**

These preliminary results indicate a strong potential to further investigate implementation of AI in the hands of patients for the reduction of the healthcare burden in primary care. Evaluating the performance and cost-effectiveness in a larger, more diverse patient population are crucial next steps.

## Introduction

General practitioners (GPs) face an increasing number of consultations for potentially cancerous skin lesions that most of the time turn out to be benign.^1^^,^^2^ This increasing workload is complicated even further by their struggle to recognize cutaneous malignancies,^3^^,^^4^ resulting in avoidable referrals of benign skin lesions to secondary care or late recognition of cutaneous malignancies.^4^, ^5^, ^6^

Over the past few years, artificial intelligence (AI)-based algorithms have been developed for detection of skin cancer. Some of these algorithms are now available on mobile phone (mHealth) applications and available to the general population or for usage by healthcare providers.^7^^,^^8^ Using simple smartphone pictures, these apps can classify suspicious skin lesions as high or low risk for skin cancer. It is important that when implemented, the accuracy of these apps is higher than the current standard of care, to reduce the risk of potential negative consequences. If such conditions are met, then implementation of such mHealth apps could be a potential solution to the high patient volume and the diagnostic challenges faced by GPs.

We envision that this technology can be employed in two distinct phases of the healthcare journey to streamline care for patients with suspicious skin lesions. First, these apps could be used by laypersons as a triaging tool prior to consulting a GP, which could potentially lead to a reduction in consultations for benign skin lesions. The second phase could be usage of an app by the GP as a diagnostic aid for assessment of suspicious skin lesions. This form of care augmented by AI could potentially increase the diagnostic accuracy of GPs and support appropriate care.^9^, ^10^, ^11^

A large prospective study is needed to investigate if implementation of AI at either point can actually improve the pathway of patients with suspicious skin lesions in primary care. However, since this has not been investigated before, it is uncertain if conducting such a study in a primary care setting is feasible. Therefore, we performed a pilot feasibility study to investigate the conditions and feasibility of a study that incorporates an AI-based app in primary care and evaluate the potential impact.

## Methods

### Study design and participants

From November 22nd, 2021 to June 9th, 2022, we conducted a within-subject pilot feasibility study with a mixed-method design to include both quantitative and qualitative data. The primary objectives of this study were to evaluate the feasibility through the inclusion and successful participation rate of patients with suspicious skin lesions and their general practitioners (GPs) (as defined in Supplemental Table S1). The first secondary objective was to identify reasons, facilitators, and barriers for successful participation. The second secondary objective was to evaluate the impact of the mHealth app for sample size calculations of the intended future study. This was done by: 1) evaluating the impact as a triaging tool in the hands of patients by specifically looking at the potential reduction of visits for benign skin lesions and the number of false negative assessments by the app, and 2) by evaluating the impact of the app as a diagnostic aid for GPs.

All patients older than 18 years who contacted their GP because of a suspicious skin lesion were eligible for inclusion. Exclusion criteria were age under 18 years, inability to give informed consent and skin lesions that had been treated or biopsied before. The study took place in three distinct GP practices in the Netherlands (in Rotterdam, Leiden and Katwijk) and lasted for 3.5 months per practice. A total of 13 GPs participated in the study.

Patients who met the eligibility criteria were offered to use an mHealth app (SkinVision, Amsterdam, the Netherlands) on their own at home or in the GP practice prior to their consultation, and were asked to fill in a questionnaire related to how this app would affect their need to visit their GP (Phase 1) (Supplemental Fig. S1). The GP would then see the patient, blinded to the outcome of the app (care as usual) after which they filled in their working diagnosis, differential diagnosis and treatment plan in the electronic health record of the patient. Immediately after the consultation, GPs were unblinded and asked whether they would change their diagnosis and/or treatment based on the app's assessments (Phase 2). If a step from either of the phases was not successfully completed, this was registered.

The electronic patient file of the GP was used to follow-up on the consultations to collect pathology reports of the suspicious skin lesions and letters from the dermatologist if a patient was referred. To verify the app's assessment, all photos made with the app were additionally assessed by a minimum of three teledermatologists, who rated each picture independently. Histopathology was used as gold standard for the final diagnosis. However, if unavailable, the clinical diagnosis of the dermatologist was used. If both of these were unavailable, the diagnosis was based on the assessment of a panel of three teledermatologists. When at least two tagged diagnoses matched, this was used as reference diagnosis. When all three tagged diagnoses did not match, the case was discussed within the research group (ASG, TS, MW) until consensus on a benign or (pre)malignant classification was reached.

Qualitative data was collected throughout the study process and analysed and reported according to the Standards of Reporting Qualitative Research (SRQR) guidelines.^12^ Audio-diaries were recorded by patients during the inclusions at the GP practice and by GPs immediately after a consult to gain a real-time in depth understanding about the experiences with the app (n = 31, total recording time of 2 h and 39 min). Additionally, the researchers (ASG, SH, JR, RL, MS) recorded detailed notes (n = 21 field observations) of the inclusion process and the interaction between patients and GPs in order to collect relevant information that could not be captured in the audio-diaries. After the inclusion period, two focus groups and one semi-structured interview were held with GPs (n = 5) and doctor's assistants (GPA) (n = 3) (total recording time of 1 h and 50 min) (Supplemental Table S2). These face-to-face sessions were held at the three GP practices to spatially remind participants of the interactions with patients in the study. To structure the focus groups and interviews, a topic guide was used (Supplemental methods). The main topics were 1) experiences during the study and perceived feasibility, 2) experiences with the app inside and outside of the study, and 3) potential for future implementation. No compensation was provided to participants. All sessions were audiotaped.

The mHealth app (SkinVision) has not been cleared by the Food and Drug Administration (FDA), but is already cleared and available to be downloaded on Android and iOS smartphones and can be used by laypersons in Europe, Australia, and New-Zealand.^13^^,^^14^ The app is registered as a CE class I-marked medical device and was validated with an estimated sensitivity of 87–95% and specificity of 70–78% to detect skin cancer.^15^^,^^16^ It uses a convolutional neural network (CNN) to classify photos as high or low risk of being a cutaneous premalignancy or malignancy and advises users with a high risk assessment to visit their GP. To ensure sufficient quality of the photo, only the front-facing camera of a phone can be used and the app automatically checks the acquisition conditions (e.g., lighting, sharpness, contrast) before allowing the photo to be taken.

The study was assessed by the Medical Ethics Committee of the Erasmus University Medical Center (MEC-2021-0254). They deemed it as not under the scope of the Medical Research Involving Human Subjects Act (WMO) and exempted it from further ethical approval. Informed consent was collected from all study participants.

### Qualitative data analysis

Recordings were transcribed verbatim into anonymized transcripts and analysed in NVivo (version 1.6.1). A thematic analysis, embedded in the constructivist paradigm, was performed using elements of grounded theory to identify the main barriers and facilitators of inclusion and participation of patients and GPs in the study. First, transcripts and observations of four cases were open and independently coded by two researchers (ASG, JR), after which they were discussed until a consensus was reached. Following this, the open codes were discussed within a research group with experienced qualitative researchers (RW, SH, TS), resulting in a preliminary list of open codes. This was repeated for the remainder of transcripts and observations in an iterative process. In the second phase, all open codes were clustered (e.g., axial coding) and categories and subcategories were defined separately by two researchers (ASG, JR). Next, these categories were discussed within the research team (RW, SH, TS) until a final consensus was reached, and the barriers and facilitators were defined.

### Statistical analysis

Descriptive statistics were used to present patient characteristics, the inclusion rate, and the participation rate. Since the aim of this study was to assess the successful inclusion rate in a given time period, no sample size calculation was done prior to the start of the study. Additionally, because completeness of data collection was also one of the study outcomes to assess successful participation, the missing data were reported in the results. A one-sample z-test for proportions was used to determine the potential reduction of patients with benign skin lesions at the GP practice and to determine whether app usage by patients led to a decrease in visits to the GP for premalignant and malignant skin lesions. To exploratory investigate the potential of the app as a diagnostic aid for future studies, we calculated the sensitivity and specificity of the app, of the GP and the GP in combination with the app based on a binary classification of the final diagnosis (benign/(pre)malignant) and diagnosis of the GP (Supplemental Table S3, Supplemental Table S4). Corresponding 95% confidence intervals (CI) were calculated. As an additional exploratory analysis, we calculated if these proportions changed when stratified for sex, self-reported skin type, GP practice, and lesion location. Statistical analyses were done in IBM SPSS Statistics for Windows, version 28.0 (IBM Corp., Armonk, NY, USA) and R statistical software (version 4.1.3).^17^

### Role of the funding source

The funder had no role in the design of the study, data analysis, data interpretation, writing of the manuscript or in the decision to publish the manuscript. All authors had full access to the data. All authors contributed to drafting of the report, read and approved the final version of the manuscript and take responsibility for its content and the final decision to submit for publication.

## Results

**Fig. 1:**
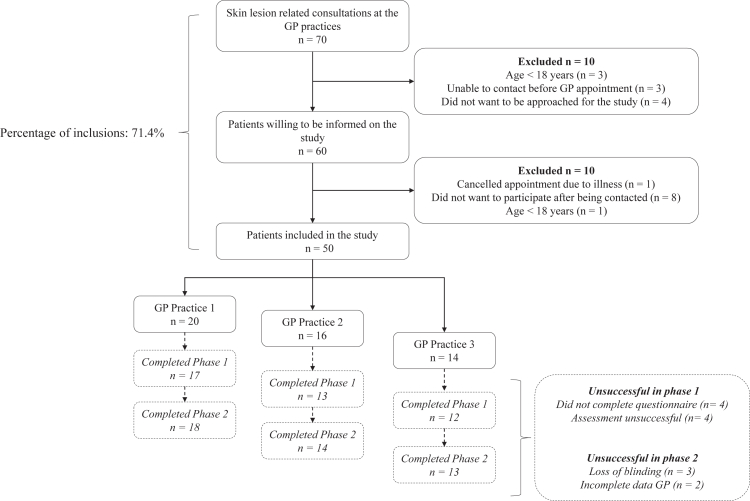
**Flowchart of the study inclusion rate of patients with suspicious skin lesions consulting one of the three GP practices between November 2021 and June 2022**. The patient trajectory from inclusion until usage of the app and completion of the questionnaire was defined as phase one. Phase two was defined as participation by the GP, which was the consult with the patient and filling in of the GP specific questions. Criteria for successful or unsuccessful participation are defined in Supplemental Table S1. Abbreviations: GP; general practitioner.

A total of 70 patients presented with a suspicious skin lesion at the GP practices during the inclusion period, of which 71.4% (n = 50) were successfully included (Fig. 1). Four patients did not want to be approached for the study, three patients could not be reached prior to the their appointment, and one did not meet eligibility criteria. Patients who were informed on the study but chose not to participate cited various reasons, including a lack of time (n = 2), considered themselves too old (n = 1), cancelled the consultation with the GP (n = 1), had a low risk rating from the app after use on own initiative, after which the patient did not see a reason to visit the GP anymore (n = 1), considered an app as too difficult and preferred to see a GP only (n = 1) and two patients refused to disclose a reason.

On average, 4–6 patients were included for the study per GP practice per month (Fig. 1). The median age of the included patients was 52 years (IQR 33.5–60.3), 64% (n = 32) were female, 90% (n = 45) had a self-reported white skin type, and 62% participated at home (Table 1). Most patients described symptoms of the skin lesion or had observed some changes in a lesion (66%) and the majority of lesions were located on the head and neck (32%) and chest and abdomen (38%).

**Table 1:** Baseline characteristics of included patients and skin lesions.

Patients	n = 50
Median age, years (IQR)	52 (33.5–60.3)
Sex, n (%)	
Male	18 (36)
Female	32 (64)
Self-reported skin type, n (%)	
White	45 (90)
Light brown	4 (8)
Dark	1 (2)
Location of participation, n (%)	
At home	31 (62)
At the GP practice	19 (38)
**Lesion characteristics**	**n = 50**

Location, n (%)	
Head and neck	16 (32)
Back	8 (16)
Chest and abdomen	19 (38)
Upper and lower extremities	7 (14)
Symptoms^a^, n (%)	
None	17 (34)
Itching	14 (28)
Changes in size, shape and/or colour	17 (34)
Pain	2 (4)
Bleeding	5 (10)
Other	3 (6)

Abbreviations: IQR; Interquartile range, GP; General practitioner.

a Numbers add up to more than 100% due to the possibility of multiple symptoms per lesion.

### Successful patient participation

84% (n = 42) of the patients successfully used the app and completed the questionnaire (Fig. 1). Less than half of the patients were able to successfully use the app on their own (44%, n = 22) and the majority needed help from the researcher or a friend/family member (48%, n = 24). In four cases, neither the patient nor the researcher was able to successfully make an assessment of the skin lesion with the app, because the lesions were located on or around the ear and covered by hair (n = 2), or were unpigmented causing the algorithm to be unable to distinguish the lesion from normal skin (n = 2).

**Fig. 2:**
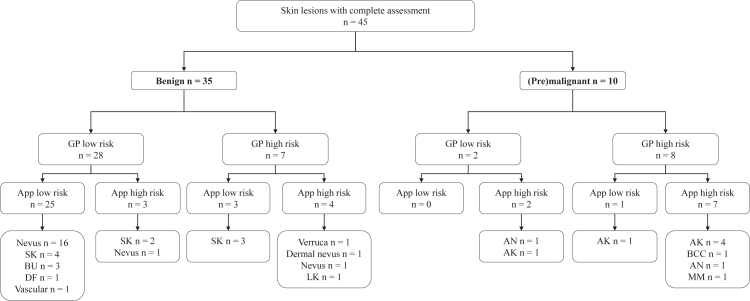
**Flowchart describing the risk classification by the general practitioner (GP) and the app in comparison to the actual final diagnosis of the lesion according to the gold standard.** Abbreviations: GP; General practitioner, SK; Seborrheic keratosis, BU; Benign unspecified, DF; Dermatofibroma, LK; Lichen planus-like keratosis, AN; Atypical nevus, AK; Actinic keratosis, BCC; Basal cell carcinoma, MM; Malignant melanoma.

There were 35 patients with a benign lesion, of which the app correctly identified 28 (specificity of 80.0% (95% CI 63.0–91.6)) as low risk. Ten patients presented with a (pre)malignancy, of which the app correctly identified 9 cases (sensitivity of 90.9% (95% CI 55.5–99.8)) as high risk (Fig. 2). These proportions were similar when stratified for sex, self-reported skin type, GP practices and location of the lesion (Supplemental Table S5). Of the patients with a benign skin lesion and a low risk rating, 54% (n = 15, Z = −33.4, p < 0.001) indicated that with this result they would be reassured to stay at home and cancel their GP visit. The one false negative rating, was a patient with an actinic keratosis who indicated they still would have visited the GP (Z = 0.318, p = 0.375).

### Successful GP participation

In 90% (n = 45) of the consultations the GP successfully participated in the study. The GPs correctly identified 8 out of 10 (sensitivity of 80.0% (95% CI 44.4–97.5)) (pre)malignant skin lesions and 28 out of 35 (specificity of 80.0% (95% CI 63.1–91.6)) benign skin lesions (Fig. 2). The GPs did not change their working diagnosis based on the app's assessment. However, in five cases the app changed their treatment plan (Supplemental Table S6). In two cases the GP and the app rated an actinic keratosis as high risk, however the app led them to be more “aggressive” in their treatment. Instead of treating with liquid nitrogen, one patient was referred to a dermatologist and in the other case a biopsy was considered. In two other cases, the GP first evaluated a lesion as low risk, but the GP changed their treatment plan because of a high risk rating from the app. In one case the GP referred the patient to a dermatologist, who diagnosed the lesion as premalignant. In the other case the GP performed an unnecessary excision of a seborrheic keratosis. Finally, in one case both the app and the GP rated a benign nevus as low risk. This low risk assessment made the GP more confident regarding the diagnosis so instead of referring the patient to a dermatologist a control appointment at the GP was planned.

The final diagnosis was based on histopathology in 20% (n = 10) of the cases, in 8% (n = 4) on the clinical diagnosis by a dermatologist and in 62% (n = 31) on the assessment of the panel of teledermatologists. Data collection was mostly complete, with only a few missing data points. In 18% (n = 9) there was no information about the patients' phone type, two patients did not indicate whether they thought the app's assessment would have influenced their decision to visit a GP, and in one case the GP did not register a differential diagnosis. In six cases, there was no teledermatology assessment because of failed photos (8%, n = 4) or because the patients did not fill in the photo identifier and could not be reached thereafter (4%, n = 2). In five cases (10%) this led to a missing final diagnosis, because the lesion was neither seen by a dermatologist nor was histopathology available.

### Barriers and facilitators for successful participation

Qualitative analysis resulted in 5 overarching themes that were divided into 7 main barriers and 4 main facilitators. All themes, barriers and facilitators and corresponding subthemes are presented in Table 2. Below we elaborate on the most important concepts regarding the feasibility of the study.

**Table 2:** Barriers and facilitators for study feasibility based on the ethnographic observations, audio-diaries, focus groups and semi-structured interview with the GP, GPA and patients.

Barriers	Facilitators
**Time and resources**	
**1. Lack of time and resources causing pressure on finishing inclusions**	**1. Availability of time, resources, and personnel**
*1.1 GP and GPA are too busy, leading to time pressure for the inclusion and obstruction of data collection*	*1.1 GP and GPA available to ensure a smooth inclusion process*
*1.2 Lack of dedicated study room for the inclusion due to busy GP practice*	*1.2 Sufficient time of the GP for a smooth inclusion and complete data collection*
**Impact of the researcher**	
**2. Influence of presence or absence of the researcher on the patient and general practitioner**	**2. Presence of the researcher during inclusions facilitates data collection**
*2.1 Presence of researcher impacts natural behaviour of the patient*	*2.1 Researcher can help making the assessment with the app*
*2.2 Presence of researcher influences care as usual by the GP*	*2.2 Researcher ensures blinding of the GP*
*2.3 Absence of the researchers leads to the patient revealing the assessment and the GP losing blinding*	
**Usage of the app**	
**3. Patient and tumour characteristics hindering inclusion**	**3. Usage of the mHealth app is an effortless process**
*3.1 Lesion characteristics leading to a difficult assessment*	*3.1 Patient is technologically engaged leading to effortless usage of the app*
*3.2 Personal insecurities slowing down the inclusion*	
**4. Technological inexperience of patients**	
*4.1 Lack of experience with the study specific technological devices*	
*4.2 General technological illiteracy*	
**Study-related information provision**	
**5. Lack in provision of study-related information**	**4. Familiarity of the general practitioner with the study, leads to an easier and quicker inclusion**
*5.1 GP and GPA experience a lack of sufficient study information*	
*5.2 Patients experienced a lack of sufficient study information*	
**Other**	
**6. Practical issues in material and research facilities**	
**7. Unforeseen circumstances leading to cancelations or delays**	

Abbreviations: GP; General practitioner, GPA; doctor's (GPs) assistant.

#### Time and resources

The first theme that was identified was time and resources. The main underlying barrier was a *lack of time and resources causing pressure on finishing inclusions,* which sometimes resulted in missing data. Personnel of the practices were often busy, which for example caused the GP to have insufficient time to record an audio-diary. The lack of a dedicated research facility also hindered inclusions.

*The availability of time, resources, and personnel* were identified as a facilitator. When there was enough time for a consultation and the GP was still on schedule, GPs were more elaborate in recording audio-diaries and reflecting on the consultation. This increased the depth of the qualitative data, which is essential to understanding the impact of the app on clinical decision making. Also, when the patient had successfully entered the questionnaire at home and already used the app, this often resulted in a simple and smooth inclusion. Finally, the help of the GPAs was essential for successful inclusion of patients.

#### Impact of the researcher

The second main theme was the impact of the researcher on the consultations and inclusion process. The main barrier that we identified was the *influence of presence or absence of the researcher on the patient and general practitioner.* For example, patients sometimes quickly gave up on making an assessment with the app. It is possible that they may have felt less pressure, and thus may have continued to try to use the app, if they had not been observed. In other cases, the GP went more elaborately through the consultation or they justified their decisions to the researcher. However, if a researcher was not present during the consultation in a few instances this led to unblinding of the GP (n = 3). Either the GP asked the patient for the result or the patient themselves accidentally revealed the app's assessment.

Additionally, *presence of the researcher during inclusions facilitates data collection.* The research personnel played an active role in contacting the patients prior to the GP visits and actively recruited them for the study. During inclusions, the researcher could prevent unblinding of the GP and make sure data collection was complete. For example, among patients that initially wanted to participate at home, 10% (n = 3 out of 31) needed help because they forgot to fill in part of the questionnaire. Presence of the researcher was also perceived as positive by the staff of the GP practice.

Another advantage was that when present, the researcher could help make the assessment with the app if required. This was needed in 6% (n = 2 out of 31) of the patients that initially participated at home, and 95% (n = 18 out of 19) of the patients that participated at the GP practice. Additionally, presence of the researcher allowed for follow-up questions on the spot, collecting more in-depth data.

#### Usage of the app

**Fig. 3:**
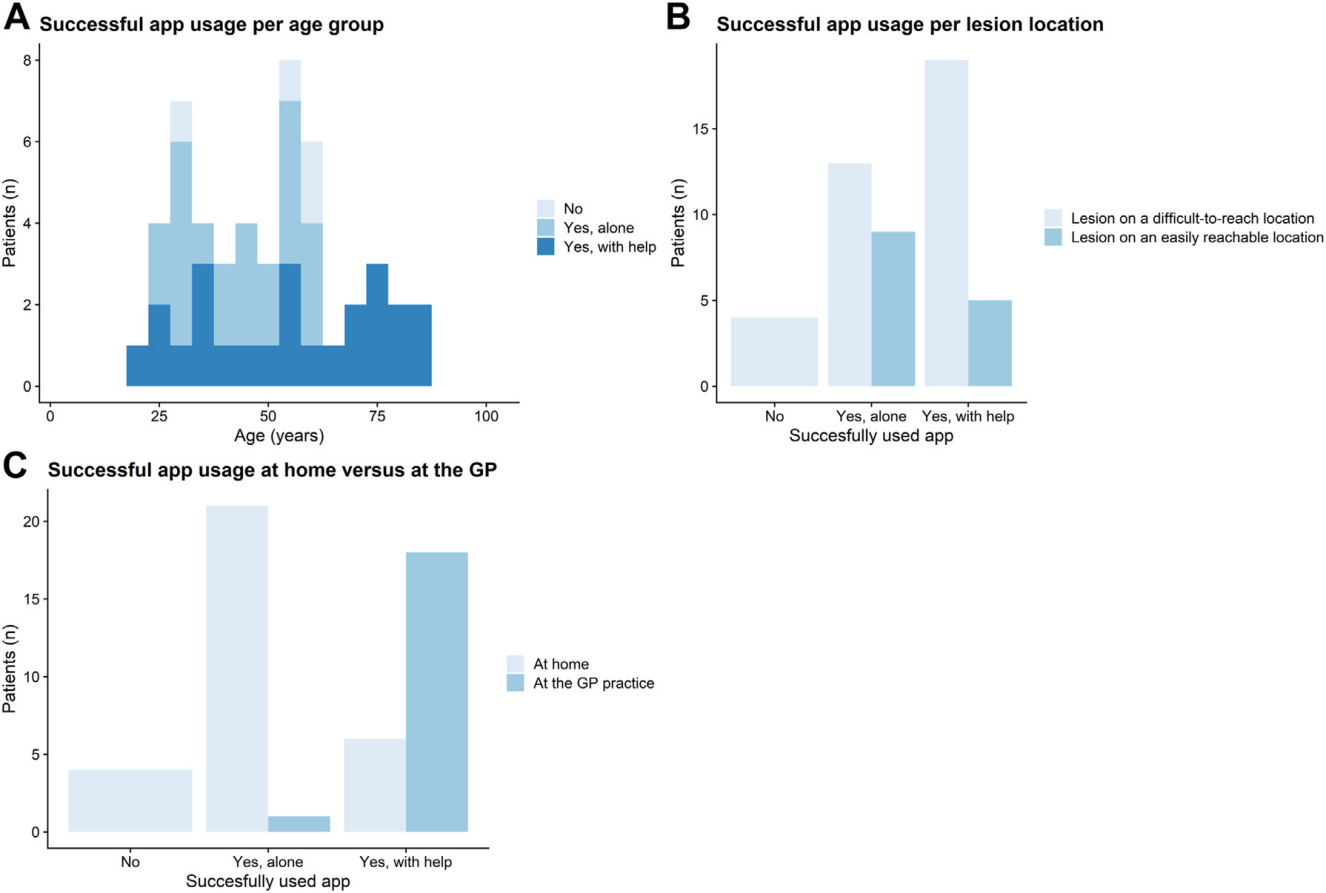
**Factors related to (un)successful usage of the app**. Abbreviation: GP; General practitioner.

Usage of the app was influenced by many factors. Patient characteristics such as *personal insecurities* or *the location of the lesion* influenced usage of the app and often caused the need for the researcher to assist. For example, when lesions were on difficult-to-reach locations, this impacted the ability of patients to use the app themselves, because they simply could not reach it. This was also reflected in the quantitative data, in the cases where patients needed help, 79% (n = 19) of the lesions were located on a difficult location on the body (Fig. 3b). Also *technological inexperience of patients* was identified as a barrier that hindered usage of the app and filling out the digital questionnaire. This was often related to lack of experience with the study specific devices or general technological illiteracy. These were influenced by insecurities of the patient or older age. However, it was usually due to a combination of all these factors and the time pressure (Fig. 3), since some older patients also succeeded in making the picture themselves.

A facilitator was when *usage of the mHealth app is an effortless process.* Especially when patients were technologically engaged this led to effortless usage of the app*.* Patients who participated at home more often succeeded in using the app on their own. (Fig. 3c).

#### Study-related information provision

Another important theme was the study-related information provision to the patients and the GP. We identified that a *lack in provision of study-related information* led to confusion and delays for both the GP, GPA and the patients and that *familiarity of the general practitioner with the study* leads to an easier and quicker inclusion.

## Discussion

This study describes the feasibility and potential of targeted implementation of an AI-based mHealth app in primary care. Studying implementation at two different moments in the healthcare pathway of people with suspicious skin lesions appears feasible and we have identified multiple barriers and facilitators that provide solid groundwork which can be incorporated in future studies. Results from the study indicate a strong potential for future research directions to focus on usage of the app as a targeted triaging tool in the hands of patients. However, a final prospective study, including a larger and more diverse group of patients and GPs, is needed to draw definite conclusions on the effect of the app on this healthcare pathway before steps towards implementation can be taken.

In this pilot feasibility study, the app reached an accuracy similar to what has been reported in validation studies.^8^^,^^15^^,^^18^ When evaluating the impact on the care pathway, we found that 54% of the patients with a benign skin lesion and a low risk rating from the app reported they would have stayed at home with this assessment and the one patient with an incorrect low risk rating from the app indicated they still would have visited the GP. Since the average Dutch GP faces around 177 consultations for benign skin lesions per year,^2^^,^^19^^,^^20^ implementation of this intervention could therefore potentially lead to a significant reduction in consultations for benign skin lesions, without the risk of increasing the workload of physicians due to false positives.^21^^,^^22^ However, this should be balanced against the risk of missing skin cancer (the false negatives) and delays in appropriate care. This is especially important for melanomas and cutaneous squamous cell carcinomas, where the 5-year survival decreases for tumors detected at a late stage.^23^^,^^24^ Therefore, a future study should additionally be powered for a non-inferiority analysis to additionally evaluate the safety of implementation in a primary care setting.

A mayor concern that needs further evaluation and development is intended use. A large part of the study population (48%) was not able to use the app on their own and required assistance from others, diminishing the potential impact of the app. This could partially be addressed by improving usability, but some obstacles are inherent to AI in general. A concern when implementing AI in clinical care is that it can lead to exclusion of patients and perpetuate inequality. For example for those with a darker skin type, as multiple studies have demonstrated that algorithms tend to underperform due to bias in training data,^25^^,^^26^ or for patients with low technological literacy who have difficulties using smartphones.^27^, ^28^, ^29^ Additionally, some lesions might even never be suitable for this technology, such as lesions that are covered by hair, lie in a body fold and are difficult to photograph or where algorithms struggle with noise removal.^15^^,^^16^^,^^30^^,^^31^ It is therefore important to be aware of these limitations and search for solutions through both algorithm improvements and ensuring that healthcare remains accessible to all patients.

Besides impact on the patient, we also evaluated how the app could assist the GP to evaluate the potential of- and possible recommendations for this future research direction. In reader studies, offering AI assistance when rating a picture of a skin lesion significantly increased the accuracy of GPs, and therefore we expected to find a similar effect in this study.^9^^,^^10^^,^^32^ However, the GPs who participated in this pilot feasibility study did not change their working diagnosis based on the app's assessment and correctly classified the majority of the benign and (pre)malignant skin lesions.^5^^,^^6^ A potential explanation for this high accuracy is that the GPs participating in this study had more affinity with dermatology. For a future study it is important to include a more diverse group of GPs who are less experienced in triaging suspicious skin lesions, since AI affects healthcare providers differently based on how experienced they are.^32^

Nevertheless, some GPs changed their treatment plan based on the app's assessment. The app only classifies a lesion as high or low risk and cutaneous premalignancies, which might sometimes warrant a more nuanced classification, are still subject to this binary classification. In this study the app classified two premalignancies as high risk, which caused the GP to unnecessarily change to a more rigorous approach. Future studies might put a special focus on this subgroup of premalignant lesions. Either by providing GPs with a more detailed explanation on how the algorithm classifies lesions or by having the algorithm classify premalignancies as a separate category.

Because this is a pilot feasibility study, the results are mainly focused on the feasibility and results that report the impact of the app are only indicative of a potential research direction and should be validated in a larger, adequately-powered study. We found that including patients for a future study is feasible. Involvement of the researcher played a very important role in the inclusion rate of patients. The research staff was actively involved in the inclusion process, called all patients themselves and attended the majority of the consultation at the GP practice. The GP did not have to spend any time actively including patients and the practice assistants who are in charge of the GPs schedule only had to ask patients whether they wanted to be contacted by a researcher. Therefore, most of the responsibility came from the researchers who had ample time for inclusions. To ensure sufficient inclusions in a future study, it is therefore recommended for a dedicated researcher to be actively involved in the inclusion process. A margin of error should also be included in the power calculations. Some barriers and facilitators were more important for the successful participation rates of patients and GPs. Facilitators such as enough time and well-explained study-related information are crucial. A lack of time and therefore increased pressure diminishes productivity, but also leads to a reduction in adherence to guidelines,^33^^,^^34^ which could impact the results of the study. Additionally, presence of the researcher can influence care as usual and the natural behaviour of GPs and patients. This is a known phenomenon called the Hawthorne effect.^35^^,^^36^ Researchers cannot always prevent this, but should be aware of this effect and use it to their advantage. Further recommendations for a future study are; i) to plan extra time for study-related consultations to ensure enough time for- and increased quality of data collection, ii) to always have a dedicated research assistant on site so that personnel of the GP practice is not burdened by the study and help can be offered when required and iii) to clearly inform all the patients and participating personnel on the study procedures prior to inclusion, to minimize the risk of unsuccessful participation or data collection in each phase.

The strength of this pilot feasibility study is the mixed methods evaluation by a multidisciplinary team, that allowed us to gain an in depth qualitative understanding of the experiences of GPs and patients with the app. Furthermore, because patients could both participate at home and at the GP practice this provided data from both settings, providing insight of intended use in both situations. A limitation of this study was that there were only ethnographic observations of app usage for patients who participated at the GP practice. A large part of this group did not participate at home because they indicated that they had difficulties using technology. Therefore, there might be a bias in observations towards barriers of app usage over facilitators. Second, not all lesions had a histopathological diagnosis and the gold standard was mostly based on the assessment of a panel of teledermatologists. However, since the accuracy of teledermatologists for diagnosing skin cancer is generally very high (sensitivity of 94.9% (95% CI 90.1%–97.4%)) we assume the risk of misdiagnosis is low.^37^ Third, most study participants (90%) had a white skin type which might not accurately reflect on the entire population. Considering that 25.2% of the Dutch population has a non-Dutch ethnicity,^38^ this is something that should be accommodated for in a future study by ensuring inclusion of a more diverse patient population in terms of ethnic background and skin types.

In conclusion, we found that including sufficient patients at a good inclusion rate for a future study is feasible. Many points for improvement were found that can be incorporated to increase the chance of successful participation by GPs and patients. This pilot feasibility study indicates potential usage of this app as a triaging tool for laypersons prior to visiting their GP. No effect was detected on the diagnostic accuracy of the GP, although there were some changes in their treatment plans. A larger prospective study with a more diverse group of patients and GPs is needed to validate these findings.

## Contributors

ASG, TS, JE, SH, PB, RW and MW contributed to the design of the study. ASG, TS, SH, JR, RL, MS, RW and MW wrote the analysis plan. ASG, TS, SH, JR, RL, MS and RW verified the underlying data. ASG, RL and MS did the statistical analysis. ASG, TS, SH, JR and RW did the qualitative analysis. ASG, TS, SH, JR, RL, MS, RW and MW interpreted the analyses. ASG wrote the first draft of the report with input from TS and MW. All authors had full access to the data. All authors contributed to drafting of the report, read and approved the final version of the manuscript and take responsibility for its content and the final decision to submit for publication.

## Data sharing statement

Data collected for this study will not be made publicly available. Study protocol, statistical analysis plan and analytic code will be shared on request for academic purposes. Proposals should be directed to m.wakkee@erasmusmc.nl. To gain access to the study protocol, statistical analysis plan and analytic code, data requestors will need to sign a data access agreement.

## Declaration of interests

The Erasmus MC Department of Dermatology has received an unrestricted research grant from SkinVision B.V. None of the authors received any direct fees for consulting or salary from the company. Tobias E Sangers declares speaker honoraria from Pfizer, Janssen-Cilag, and UCB. There are no other declarations of interest.

## Appendix ASupplementary data

Supplementary Methods, Supplementary Figs. S1 and Supplementary Table S1–S6

Standards for Reporting Qualitative Research
