# Supplementary material for: Artificial intelligence in mobile health for skin cancer diagnostics at home (AIM HIGH): a pilot feasibility study
Source: eClinicalMedicine. 2023 May 25;60:102019. doi: 10.1016/j.eclinm.2023.102019 (PMC10227364; doi:10.1016/j.eclinm.2023.102019)
Supplement: Supplementary Methods, Supplementary Figs. S1 and Supplementary Table S1–S6 [file mmc1.pdf]

**Supplemental material**

**Supplemental methods**

Classification of difficult-to-reach location

Lesions were classified as on a difficult-to-reach location or an easily reachable location. A location was difficult to reach if it was on the back of the thighs, the buttocks, the back, the neck, the head, the upper region of the thorax and the backside of the upper arms and shoulders.

Topic list focus groups and semi-structured interview

We present a summarized versions of the topic list for the focus groups and semi-structured interview:

- How do GPs and GP assistants reflect on participating in the study
- How do GPs and GP assistants reflect on the impact of the app inside and outside of the study? Does the fact that they're participating in the study impact other consultations, even when they aren't using the app?
- What do GPs think about using the app outside of the study? Will they recommend the app to patients or friends outside of the study?
- Visceral experience of using the app in patient interaction
- Emotional experience of using the app in patient interaction
- Change in decision-making as a result of using the app
- Change in relationship with patient when mediated through technology
- Change in practices when mediated through the app

**Supplemental figure 1.** Study design.

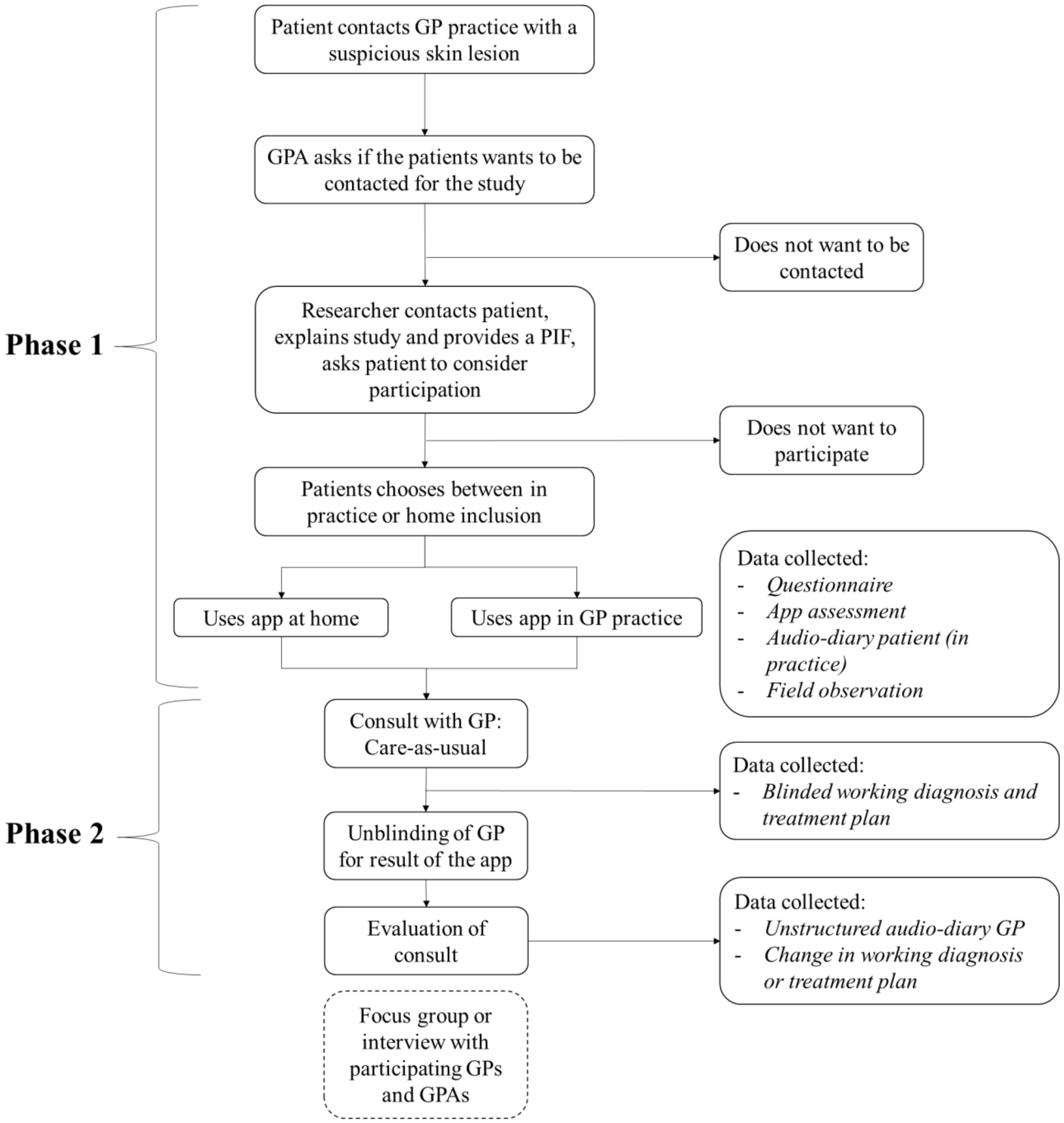


Abbreviations: GP; general practitioner, GPA; doctor's (GPs) assistant, PIF; Patient information folder.

**Supplemental table 1** Criteria for successful participation by the patient and general practitioner (GP).

| **Phase 1: Patient**<br>Usage of the app and filling in of the questionnaire | **Phase 2: General practitioner**<br>Consultation by the general practitioner and filling in of the questionnaire |
|---|---|
| 1.Filled in entire questionnaire | 1.Blinding successful |
| 2.App assessment successful (with or without help) | 2.Filled in a blinded and unblinded diagnosis and treatment plan |

Participation per phase was successful if both criteria were met.

**Supplemental table 2.** Description of the collected qualitative data

| **Qualitative data** | |
|---|---|
| Field observations made by the researchers | |
| Of the patient (n) | 19 |
| Of a consultation by the GP (n) | 21 |
| Audio-diaries | |
| Recorded by the patient (n) | 8 |
| Recorded by the GP (n) | 23 |
| Focus groups | |
| Participants of focus group 1 | 2 GPs, 2 GPAs |
| Participants of focus group 2 | 2 GPs, 1 GPA |
| Semi-structured interview | |
| Participants | 1 GP |

Abbreviations: GP; general practitioner, GPA; doctors assistant.

**Supplemental table 3.** Binary classification of diagnoses for calculation of the sensitivity and specificity.

| **Diagnosis** | **Classification** |
|---|---|
| LS/SL | Low |
| Dermatofibroma | Low |
| SK | Low |
| Vascular (hemangioma, pyogenic granuloma, telangiectasia) | Low |
| Benign nevus | Low |
| Lichen planus-like keratosis | Low |
| Atypical nevus if considered clinically benign | Low |
| Atypical nevus if considered malignant | High |
| BCC | High |
| cSCC | High |
| MM | High |
| LM | High |
| BD | High |
| AK | High |
| Keratoacanthoma | High |

Classification reflects how the final diagnosis was made into a binary category. Abbreviations: LS; lentigo simplex, SL; solar lentigo, SK; seborrheic keratosis, BCC; basal cell carcinoma, cSCC; cutaneous squamous cell carcinoma, MM; malignant melanoma, LM; lentigo maligna, BD; Bowens disease, AK; actinic keratosis.

**Supplemental table 4.** Binary classification of the assessment of the GP based on a combination of the working diagnosis, differential diagnosis and treatment plan.

| Working diagnosis | Differential diagnosis | Treatment plan | Classification GP |
|---|---|---|---|
| Sebaceous cyst | Only benign | Excision or expectant | Low |
| Dermatofibroma | Only benign | Expectant | Low |
| | Only benign | Biopsy, excision or referral* | High |
| | Includes malignancy | Expectant | Low |
| | Includes malignancy | Biopsy, excision or referral* | High |
| Halo nevus | Only benign | Expectant | Low |
| | Only benign | Biopsy, excision or referral* | High |
| | Includes malignancy | Expectant | Low |
| | Includes malignancy | Biopsy, excision or referral* | High |
| SK | Only benign | Expectant | Low |
| | Only benign | Biopsy, excision or referral* | High |
| | Includes malignancy | Expectant | Low |
| | Includes malignancy | Biopsy, excision or referral* | High |
| Verruca vulgaris | Only benign | Expectant | Low |
| | Only benign | Biopsy, excision or referral* | High |
| | Includes malignancy | Expectant | Low |
| | Includes malignancy | Biopsy, excision or referral* | High |
| Solar lentigo | Only benign | Expectant | Low |
| | Only benign | Biopsy, excision or referral* | High |
| | Includes malignancy | Expectant | Low |
| | Includes malignancy | Biopsy, excision or referral* | High |
| Benign nevus | Only benign | Expectant | Low |
| | Only benign | Biopsy, excision or referral* | High |
| | Includes malignancy | Expectant | Low |
| | Includes malignancy | Biopsy, excision or referral* | High |
| Atypical nevus | Only benign | Expectant | Low |
| | Only benign | Biopsy, excision or referral* | High |
| | Includes malignancy | Expectant | Low |
| | Includes malignancy | Biopsy, excision or referral* | High |
| BCC | NA | Biopsy, excision or referral | High |
| cSCC | NA | Biopsy, excision or referral | High |
| MM | NA | Biopsy, excision or referral | High |

Classification reflects how the working diagnosis of the GP in combination with the differential diagnosis and treatment plan was made into a binary high or low risk for calculations of the sensitivity and specificity. There were no other working diagnoses by the GP then the abovementioned options. Abbreviations: SK; seborrheic keratosis, BCC; basal cell carcinoma, cSCC; cutaneous squamous cell carcinoma, MM; malignant melanoma, LM; lentigo maligna, BD; Bowens disease, AK; actinic keratosis, NA; Not applicable.

*If send for pathological examination and not for cosmetic reasons

**Supplemental table 5.** Exploratory subgroup analyses of the accuracy of the app when stratified for sex, self-reported skin type, GP practice, lesion location and difficult to reach locations.

| | **Proportion of correctly identified benign lesions, % (n/N)** | **Proportion of correctly identified (pre)malignant lesions, % (n/N)** |
|---|---|---|
| Sex | | |
| Male | 92.3 %(12/13) | 100% (4/4) |
| Female | 72.7% (16/22) | 83.3% (5/6) |
| Self-reported skin type | | |
| White | 78.1% (25/32) | 88.9% (8/9) |
| Light brown | 100% (2/2) | 100% (1/1) |
| Dark | 100% (1/1) | NA (0/0) |
| GP practice | | |
| Practice 1 | 76.9% (10/13) | 100% (4/4) |
| Practice 2 | 75% (9/12) | 100% (2/2) |
| Practice 3 | 90% (9/10) | 75% (3/4) |
| Lesion location | | |
| Head and neck | 80% (4/5) | 83.3% (5/6) |
| Back | 50% (3/6) | 100% (2/2) |
| Chest and abdomen | 88.9% (16/18) | 100% (1/1) |
| Upper and lower extremities | 83.3% (5/6) | 100% (1/1) |
| Difficult to reach location | | |
| Yes | 72.7% (16/22) | 88.9% (8/9) |
| No | 92.3 % (12/13) | 100% (1/1) |

Abbreviations: GP; general practitioner, NA; Not applicable.

**Supplemental table 6**. Changes in treatment of the general practitioner based on the app's assessment.

| Final diagnosis | GP Blinded | App | Treatment blinded | New treatment plan |
|---|---|---|---|---|
| AK | High | High | $N_2$ and control appointment | Control and consider biopsy |
| AK | High | High | $N_2$ | Referral dermatologist |
| AK | Low | High | Expectant | Biopsy* |
| SK | Low | High | Expectant, possibly $N_2$ | Excision |
| Nevus | Low | Low | Referral | Control appointment |

Table describing the impact of the assessment of the app on the treatment plan of the general practitioner. Abbreviations: GP; general practitioner, SK; seborrheic keratosis, AK; actinic keratosis, $N_2$; treatment with liquid nitrogen.

*GP initially wanted to perform a biopsy. However, on request of the patient referred them to a dermatologist so the dermatologist could do the biopsy
