# Supplementary material for: Artificial intelligence in mobile health for skin cancer diagnostics at home (AIM HIGH): a pilot feasibility study
Source: eClinicalMedicine. 2023 May 25;60:102019. doi: 10.1016/j.eclinm.2023.102019 (PMC10227364; doi:10.1016/j.eclinm.2023.102019)
Supplement: Standards for Reporting Qualitative Research [file mmc2.pdf]

Standards for Reporting Qualitative Research (SRQR)*

http://www.equator-network.org/reporting-guidelines/srqr/

Page/line no(s).

Title and abstract

**Title** - Concise description of the nature and topic of the study Identifying the study as qualitative or indicating the approach (e.g., ethnography, grounded theory) or data collection methods (e.g., interview, focus group) is recommended	Page 1, line 1
**Abstract** - Summary of key elements of the study using the abstract format of the intended publication; typically includes background, purpose, methods, results, and conclusions	Page 2, lines 1 to 27

Introduction

**Problem formulation** - Description and significance of the problem/phenomenon studied; review of relevant theory and empirical work; problem statement	Page 3 lines 2 to 13
**Purpose or research question** - Purpose of the study and specific objectives or questions	Page 3 lines 14 to 24

Methods

**Qualitative approach and research paradigm** - Qualitative approach (e.g., ethnography, grounded theory, case study, phenomenology, narrative research) and guiding theory if appropriate; identifying the research paradigm (e.g., postpositivist, constructivist/ interpretivist) is also recommended; rationale**	Page 4 lines 3 to 4<br>Page 5 lines 27 to 29
**Researcher characteristics and reflexivity** - Researchers' characteristics that may influence the research, including personal attributes, qualifications/experience, relationship with participants, assumptions, and/or presuppositions; potential or actual interaction between researchers' characteristics and the research questions, approach, methods, results, and/or transferability	Page 5 lines 1 to 7<br>Page 5 lines 29 to 36
**Context** - Setting/site and salient contextual factors; rationale**	Page 4 lines 13 to 17<br>Page 5 lines 2 to 12
**Sampling strategy** - How and why research participants, documents, or events were selected; criteria for deciding when no further sampling was necessary (e.g., sampling saturation); rationale**	Page 4 lines 13 to 17<br>Page 4 line 36 to page 5 line 5
**Ethical issues pertaining to human subjects** - Documentation of approval by an appropriate ethics review board and participant consent, or explanation for lack thereof; other confidentiality and data security issues	Page 5 lines 21 to 24
**Data collection methods** - Types of data collected; details of data collection procedures including (as appropriate) start and stop dates of data collection and analysis, iterative process, triangulation of sources/methods, and modification of procedures in response to evolving study findings; rationale**	Page 4 lines 3 to 4<br>Page 4 lines 18 to 25<br>Page 4 line 35 to page 5 line 10<br>Supplemental table 2

**Data collection instruments and technologies** - Description of instruments (e.g., interview guides, questionnaires) and devices (e.g., audio recorders) used for data collection; if/how the instrument(s) changed over the course of the study	Page 4 line 36 to page 5 line 5<br>Page 5 line 10<br>Supplemental methods
**Units of study** - Number and relevant characteristics of participants, documents, or events included in the study; level of participation (could be reported in results)	Page 7 line 2 to 14<br>Supplemental table 2
**Data processing** - Methods for processing data prior to and during analysis, including transcription, data entry, data management and security, verification of data integrity, data coding, and anonymization/de-identification of excerpts	Page 5 lines 1 to 3<br>Page 5 lines 26 to 27
**Data analysis** - Process by which inferences, themes, etc., were identified and developed, including the researchers involved in data analysis; usually references a specific paradigm or approach; rationale**	Page 5 lines 26 to 36
**Techniques to enhance trustworthiness** - Techniques to enhance trustworthiness and credibility of data analysis (e.g., member checking, audit trail, triangulation); rationale**	Page 5 lines 29 to 36

Results/findings

**Synthesis and interpretation** - Main findings (e.g., interpretations, inferences, and themes); might include development of a theory or model, or integration with prior research or theory	Page 8 line 16 to page 9 line 30
**Links to empirical data** - Evidence (e.g., quotes, field notes, text excerpts, photographs) to substantiate analytic findings	Page 9 lines 2 to 5<br>Page 9 Lines 9 to 10<br>Page 9 lines 13 to 14<br>Page 9 lines 20 to 22<br>Page 9 lines 25 to 27<br>Page 9 lines 29 to 30

Discussion

**Integration with prior work, implications, transferability, and contribution(s) to the field** - Short summary of main findings; explanation of how findings and conclusions connect to, support, elaborate on, or challenge conclusions of earlier scholarship; discussion of scope of application/generalizability; identification of unique contribution(s) to scholarship in a discipline or field	Page 10 line 1 to page 11 line 34
**Limitations** - Trustworthiness and limitations of findings	Page 11 line 38 to page 12 line 9

Other

**Conflicts of interest** - Potential sources of influence or perceived influence on study conduct and conclusions; how these were managed	Page 13 lines 16 to 19
**Funding** - Sources of funding and other support; role of funders in data collection, interpretation, and reporting	Page 13 lines 16 to 19

*The authors created the SRQR by searching the literature to identify guidelines, reporting standards, and critical appraisal criteria for qualitative research; reviewing the reference lists of retrieved sources; and contacting experts to gain feedback. The SRQR aims to improve the transparency of all aspects of qualitative research by providing clear standards for reporting qualitative research.

**The rationale should briefly discuss the justification for choosing that theory, approach, method, or technique rather than other options available, the assumptions and limitations implicit in those choices, and how those choices influence study conclusions and transferability. As appropriate, the rationale for several items might be discussed together.

Reference:

O'Brien BC, Harris IB, Beckman TJ, Reed DA, Cook DA. **Standards for reporting qualitative research: a synthesis of recommendations.** *Academic Medicine*, Vol. 89, No. 9 / Sept 2014
DOI: 10.1097/ACM.0000000000000388
